# ORAL CONCURRENT SESSION 8: Fetus and Fetal Intervention

**DOI:** 10.1002/pmf2.12019

**Published:** 2025-01-05

Abstracts 86 – 94

SATURDAY

February 1, 2025

8:00 AM – 10:15 AM

Aurora Ballroom B

MODERATORS

Juan Gonzalez, MD, MS, PhD

Bettina Paek, MBA, MD

